# Outpatient palliative care during the COVID-19 pandemic: a retrospective single centre analysis in Germany

**DOI:** 10.1186/s12904-022-01035-x

**Published:** 2022-08-12

**Authors:** Jonas Behnke, Philipp Friedrich Arndt, Michael John Cekay, Daniel Berthold, Birgit Herentin, Rio Dumitrascu, Ulf Sibelius, Bastian Eul

**Affiliations:** Department of Internal Medicine IV, Justus-Liebig-University, Universities of Giessen and Marburg Lung Centre (UGMLC), Klinikstrasse 33, 35392 Giessen, Germany

**Keywords:** COVID-19, Outpatient palliative care (OPC), Advanced care planning (ACP), Cancer

## Abstract

**Background:**

The coronavirus disease 2019 (COVID-19) pandemic has challenged health care systems worldwide. In Germany, patients in a palliative care setting have the opportunity to receive treatment by a specialised mobile outpatient palliative care team (OPC). The given retrospective single centre analysis describes the use of OPC structures for terminally ill COVID-19 patients during the height of the pandemic in Germany and aims to characterise this exceptional OPC patient collective.

**Methods:**

First, death certificates were analysed in order to collect data about the place of death of all deceased COVID-19 patients (*n* = 471) within our local governance district. Second, we investigated whether advance care planning structures were established in local nursing homes (*n* = 30) during the height of the COVID-19 pandemic in 2020. Third, we examined patient characteristics of COVID-19 negative (*n* = 1579) and COVID-19 positive (*n* = 28) patients treated by our tertiary care centre guided OPC service.

**Results:**

The analysis of death certificates in our local district revealed that only 2.1% of all deceased COVID-19 patients had succumbed at their home address (*n* = 10/471). In contrast, 34.0% of COVID-19 patients died in nursing homes (*n* = 160/471), whereas 63.5% died in an inpatient hospital setting (*n* = 299/471). A large proportion of these hospitalised patients died on non-intensive care unit wards (38.8%). Approximately 33.0% of surveyed nursing homes had a palliative care council service and 40.0% of them offered advance care planning (ACP) structures for their nursing home residents. In our two OPC collectives we observed significant differences concerning clinical characteristics such as the Index of Eastern Cooperative Oncology Group [ECOG] (*p* = 0.014), oncologic comorbidity (*p* = 0.004), as well as referrer and primary patient location (*p* = 0.001, *p* = 0.033).

**Conclusions:**

Most COVID-19 patients in our governance district died in an inpatient setting. However, the highest number of COVID-19 patients in our governance district who died in an outpatient setting passed away in nursing homes where palliative care structures should be further expanded. COVID-19 patients who died under the care of our OPC service had considerably fewer oncologic comorbidities. Finally, to relieve conventional health care structures, we propose the expansion of established OPC structures for treating terminally ill COVID-19 patients.

**Supplementary Information:**

The online version contains supplementary material available at 10.1186/s12904-022-01035-x.

## Background

The COVID-19 pandemic has caused excessive demands on health care systems in many countries around the world [1–3]. Large numbers of mostly elderly and multi-morbid COVID-19 patients particulary challenged the clinical supply structures in Germany. An ageing society and demographic change amplified this development. During the last several years, palliative care medicine has gained importance in the treatment of various terminal diseases [4]. Palliative care can consist of outpatient and inpatients services [5, 6]. Inpatient palliative care may be provided through a specialised palliative clinical ward, hospice or a supporting palliative care service within established hospital structures. In Germany, all nursing home residents have the right to consult a specialised palliative care service as stated in the hospice and palliative care law in the German Social Law Order Book V (SGB V). Here, a designated member of staff supporting palliative care interests for the nursing home residents should practice a palliative care council service. The public health insurance system covers the costs for this service.

Outpatient palliative care can be a valuable alternative, especially for patients who refuse hospitalisation or intensive care treatment. In this case, every terminally ill patient in Germany has the right to receive treatment at home through a specialised OPC service (SGB V). Such outpatient teams consist of nurses and physicians specifically trained in palliative care. OPC aims to achieve optimal palliative symptom control in end stage diseases and avoid hospitalisation in accordance with patient-will. Most patients under palliative care suffer from terminal stage cancer [2].

Interestingly, critically ill COVID-19 patients regularly present with respiratory symptoms similar to those of terminal stage cancer patients, even though COVID-19 patients do not necessarily have life threatening comorbidities. Such symptoms may include dyspnea, agitation or anxiety [7–10]. Death rates are highest among patients with pre-existing conditions [11]. Most patients who died of COVID-19 initially presented with a variety of illnesses prior to their infection and typically advanced age. This makes COVID-19 a new challenge in the field of palliative care medicine [12]. Meanwhile, there is an increasing number of elderly and comorbid patients in Germany who refuse intensive care treatment or even hospitalisation [13]. Therefore, OPC can be considered for those patients who likely would not benefit from intensive care treatment such as elderly patients with COVID-19 and other untreatable terminal diseases.

A fundamentalpillar of every palliative care treatment is the informed consent of a patient. However, rapid clinical deterioration for example caused by COVID-19 can render end of life decisions in flawless accordance with patient will more difficult or even impossible [14, 15]. Pre-emptive implementation of ACP structures may help to reduce medical decisions that are not in accordance to patient wishes. Moreover, ACP implementation is also recommended by various medical societies [16].

To explore the role of palliative care medicine during the height of the COVID-19 pandemic in Germany, we investigated the place of death of all deceased COVID-19 patients in our local governance district. In addition, we analysed existing palliative care structures in nursing homes and described characteristics of COVID-19 patients under the care of our OPC service.

## Methods

To investigate palliative care structures in an outpatient setting we conducted qualitative telephone surveys at 30 nursing homes in our district,interviewing their respective directors or heads of nursing management. All interviews were carried out during December 2020. Interviews were conducted as surveys in a semi-structured way and were protocolled selectively. The interviews were evaluated with qualitative category formation and frequency analysis in an exploratory manner. For an undistorted and unbiased impression, we protocolled all interviews anonymised. As a part of the survey, we asked the respective members of nursing home staff to answer short questions about the availability of palliative care measures and ACP structures within their institutions.

**Fig. 1:**
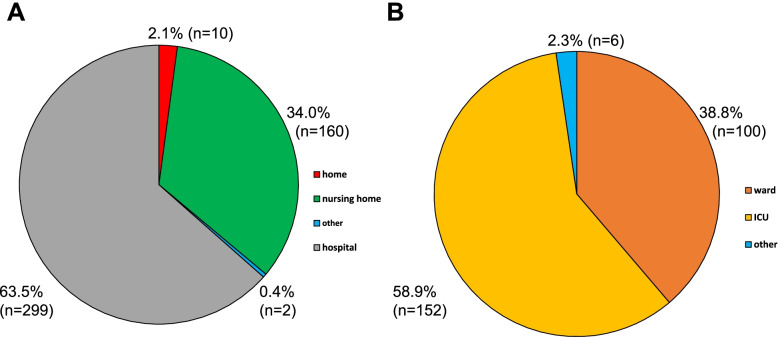
Place of death. **A** Distribution of locations of death of confirmed COVID-19 patients in the governance district of Giessen from November 2020 until April 2021 (*n* = 471). Deaths were registered by analysing all certificates of death in the district of Giessen. **B** Intrahospital locations of death of all COVID-19 deaths in the district of Giessen from November 2020 until April 2021 (*n* = 258, *n* = 41 could not be assigned). % (n)

Furthermore, we also explored a possible need for palliative care structures by analysing all death certificates of COVID-19 patients in our governance district from November 2020 until April 2021. These legal death certificates are routinely collected by the German health department and were analysed retrospectively by a qualified physician at the department. Every death certificate documents the cause and place of death and must legally be issued by a medical doctor. Intra- and extra-hospital places of death were registered and are shown in Fig. 1.

We further enrolled1607 individuals in our mono-centre retrospective register trial. A total of 28 patients in this trial had a confirmed COVID-19 diagnosis, while 1579 did not. Of the non-COVID-19 collective, 745 patients were treated in2019 and 834 patients were treated in 2020. The COVID-19 positive patients were enrolled in the OPC service from 1^st^ of December 2020 until the 31^st^ of January 2021. All patients were treated by our university hospital (tertiary care centre) OPC service. OPC patients were not routinely tested for COVID-19. Because of increasing numbers of patients in our OPC collective in 2020 the average length of treatment of the OPC collective of 2019 was compared to the OPC collective of 2020 to evaluate indirect effects of the COVID-19 pandemic (Table 1). All patient data was analysed retrospectively using routine patient records. Patient data from the OPC service was stored and retrieved from Pallidoc®-software, the routine clinical documentation program of our OPC service. A qualified palliative care nurse and a physician routinely assessed all investigated parameters in Table 2 at patient admission. Further inpatient data were gathered from the routine patient documentation system MEONA®-software of our university hospital. We collected and transferred all patient data to a database for respective calculations.,We statistically analysed all data using Student´s T-Test and Chi-square test. SPSS®-version 28 was used for all calculations.

**Table 1:** COVID-19 negative OPC patient collectives of 2019 and 2020

Item		Year treated by OPC service				
		**2019**		**2020**		
		**n (%)**	**Avg. ± SD**	**n (%)**	**Avg. ± SD**	*p* value^*^
**Gender**	**Male**	362 (48.6%)		419 (50.2%)		0.513^#^
	**Female**	383 (51.4%)		415 (49.8%)		
**Age (years)**		745	76.52 ± 12	834	77.55 ± 12	0.097^+^
**Days treated by OPC**		745	48.89 ± 83	834	44.39 ± 80	0.280^+^

^*^T-test^+^ or Chi-square test^#^; Plus–minus values are means (average, Avg.) ± standard deviation (SD)

**Table 2:** Description and comparison of COVID-19-confirmed patients and COVID-19-non-confirmed patients

Item		COVID Status				
		COVID infection not confirmed		COVID infection confirmed		
		n (%)	Avg. ± SD	n (%)	Avg. ± SD	*p* value*
Year in treated by OPC service	2019	745 (47.2%)		0 (0.0%)		< 0.001^#^
	2020	834 (52.8%)		28 (100%)		
Gender	Male	781 (49.5%)		12 (42.9%)		0.569^#^
	Female	798 (50.5%)		16 (57.1%)		
Age		1579	77.06 ± 12	28	81.00 ± 12	0.092^+^
Days treated by OPC service		1579	46.52 ± 82	28	23.32 ± 34.8	0.130^+^
ACP	No	616 (39.0%)		7 (25.0%)		0.171^#^
	Yes	963 (61.0%)		21 (75.0%)		
Legal Guardian	No	1275 (80.7%)		26 (92.9%)		0.143^#^
	Yes	304 (19.3%)		2 (7.1%)		
Referrer	University hospital	273 (17.3%)		6 (21.4%)		< 0.001^#^
	General practitioner	490 (31.0%)		21 (75.0%)		
	Other hospital	221 (14.0%)		1 (3.6%)		
	Nursing home or hospice	131 (8.3%)		0 (0.0%)		
	Oncologist	26 (1.6%)		0 (0.0%)		
	Relative	146 (9.2%)		0 (0.0%)		
	Other	292 (18.5%)		0 (0.0%)		
Care level by medical insurance	0	404 (25.7%)		7 (25.0%)		0.169^#^
	1	43 (2.7%)		0 (0.0%)		
	2	348 (22.1%)		2 (7.1%)		
	3	374 (23.7%)		8 (28.6%)		
	4	254 (16.1%)		5 (17.9%)		
	5	152 (9.7%)		6 (21.4%)		
Primary location	At home	1014 (64.2%)		14 (50.0%)		0.033^#^
	Nursing home	370 (23.4%)		13 (46.4%)		
	Hospice	188 (11.9%)		1 (3.6%)		
	Other	7 (0.4%)		0 (0.0%)		
ECOG	0	1 (0.1%)		0 (0.0%)		0.014^#^
	1	70 (4.4%)		0 (0.0%)		
	2	424 (26.9%)		3 (10.7%)		
	3	822 (52.2%)		14 (50.0%)		
	4	259 (16.4%)		11 (39.3%)		
Oncologic comorbidity	No	527 (33.4%)		17 (60.7%)		0.004^#^
	Yes	1052 (66.6%)		11 (39.3%)		
Endpoint	Stabilised	265 (16.9%)		11 (39.3%)		0.003^#^
	Deceased	1130 (72.2%)		17 (60.7%)		
	Hospital admission	171 (10.9%)		0 (0.0%)		
Emergency phone calls	No	755 (47.8%)		14 (50.0%)		0.851^#^
	Yes	824 (52.2%)		14 (50.0%)		
Emergency home visits	No	993 (62.9%)		18 (64.3%)		0.999^#^
	Yes	586 (37.1%)		10 (35.7%)		

^*^T-test^+^ or Chi-square test^#^; Plus–minus values are means (average, Avg.) ± standard deviation (SD)

## Results

Staff at 30 nursing homes were interviewed as described above, and 33.0% of these homes had an available palliative care council service. At the same time, ACP structures for nursing home residents were present in 40.0% of 25 homes. Respective data from the five remaining nursing homes were not available.

Figure 1 shows the placesof death of all deceased COVID-19 patients from our governance district from November 2020 until April 2021. Of 471 patients in our governance district Giessen, 63.5% died in a hospital setting (*n* = 299), 34.0% at a nursing home (*n* = 160) and only 2.1% at home (*n* = 10). The majority of hospitalised patients died in an intensive care unit (ICU) (58.9%, *n* = 152). In contrast, 38.8% of 258 hospitalised patients died on a general ward (*n* = 100).

In 2019 745 patients were treated by our OPC service. In 2020 numbers rose to 862 patients. As shown in Table 1 there is no significant difference in number of treatment days for patient collectives of both years (*p* = 0.28). Furthermore, there was no statistical difference concerning age (*p* = 0.097) orgender (*p* = 0.513).

In addition, we compared the OPC COVID-19 confirmed patient collective during the peak of the COVID-19 pandemic in Germany with the COVID-19 non-confirmed OPC collectives of the years 2019 and 2020 (Table 2). The non-confirmed COVID-19 collective was under the care of the OPC service during 2019 and 2020. When comparing both groups we found a balanced gender distribution (*p* = 0.569). The average age of the non-confirmed COVID-19 collective was 77.06 (± 12) and 81.00 (± 12) for the COVID-19 confirmed collective (p = 0.092). No statistical difference was observed for the number of treatment days by the OPC service (*p* = 0.130).

Table 2 summarizes the clinical baseline characteristics. Significant differences in both collectives were revealed concerning referrer (*p* = 0.001), primary location at patient admission to the OPC service (*p* = 0.033), ECOG (*p* = 0.014), oncologic comorbidity (p = 0.004) and endpoint-status (*p* = 0.003). When comparing the COVID-19 patient collective to our regular OPC collective, we observed significantly more patients with ECOG-status 4 (16.4% vs. 39.3%). Neither the collective differed in ACP (*p* = 0.171) and the presence of a legal guardian (*p* = 0.143), nor in degree of care categorised by their health insurance (*p* = 0.169). The number of emergency phone calls and visits in both groups were nearly equal (*p* = 0.851 and *p* = 0.999).

## Discussion

COVID-19 has the highest mortality among elderly patients and those with pre-existing conditions [17]. Considering the rapidly ageing western societies, especially in Germany, there is a strong demand for palliative care structures. The number of patients under the care of our OPC service increased by 89 patients between 2019 and 2020. Therefore we calculated the average treatment days for both collectives without finding a statistically significant difference. We conclude the growing number of patients is likely not an indirect effect of the COVID-19 pandemic due to patient decisions to evade hospital admissions, but rather reflects the rising demand for palliative care in general (Table 1).

Historically, palliative care mainly focused on inpatient care such as hospice or specialised clinical palliative care wards. However, due to demographic change and medical progress a rising number of multi-morbid patients live in non-hospital institutions such as nursing homes. At the beginning of the pandemic, this patient collective in particular was in danger of contracting and dying of COVID-19 [18]. In many cases, critically ill nursing home residents with COVID-19were admitted to our university hospital for further care. Many of these patients were not treated on our intensive care unit and died subsequently in one of the designated general care COVID-19 wards (Fig. 1B).

The outpatient COVID-19 patient collective we treated included 28 patients (Table 2). Only 10 COVID-19 patients in the district of Giessen died at their homes. However, all of them were treated by our OPC service. The fact that only 2.1% of COVID-19 patients died at their homes stands in stark contrast to regularly reported patient wishes mentioned in the literature, in which approximately 60.0% of all patients hope to die at home [19, 20]. This might suggest the difficulty of making adequate end-of-life-decisions for rapidly deteriorating COVID-19 patients who were previously in a relatively good or at least stable health condition. Earlier studies have shown that COVID-19 patients often are clinically stable and then deteriorate rapidly [21]. This is highlighted by our finding, that there are significantly more patients categorised as ECOG-status 4 in the OPC COVID-19 patient collective, than in the OPC non-COVID-19 patient collective. Furthermore, 46.4% of COVID-19 patients were treated at nursing homes and 21.4% had the highest degree of care possibly assigned by their health insurance. Even though the distribution of the degree of care did not statistically differ between both groups, all these parameters indicate that most of the COVID-19 collective consisted of multi-morbid patients. However, a limitation of this study was the considerably low number of COVID-19 positive patients in our OPC service and the lack of standardised testing in patients without symptoms (debatable number of undetected COVID-19 cases among the non-confirmed COVID-19 collective). Furthermore, the number of hospitalised and deceased COVID-19 patients started to decline rapidly after the implementation of the German vaccination program in December 2020 (weekly status report of Robert Koch Institute). This was especially true for elderly and multi-morbid patients as their vaccinations were prioritized. This also explains the difference in observation time for the confirmed COVID-19 and the non-confirmed patient collective, as the vaccinations started to prevent severe COVID-19 infections.

In addition, no hospital admissions were observed in the confirmed COVID-19 OPC collective. Nevertheless, it is unclear whether well prepared ACP planning and sufficient symptom control or reduced status of health are responsible for this finding. Multimorbidity accompanied by low mobility in this collective also could offer a possible explanation for the lack of hospital admissions.

Previous studies have shown that silent hypoxia precedes respiratory failure and ultimately cardiac arrest in COVID-19 patients [21]. Therefore, OPC service providers should be well trained in palliative respiratory symptom control. In our OPC patient collective the number of emergency home visits and emergency phone calls were not significantly different among the non-COVID-19 and the COVID-19 collective (Table 2). Whether this was caused by rapid deterioration of COVID-19 patients or simply reflects equivalent symptom control in both collectives has to be further explored in order to understand the needs of palliative COVID-19 patients. Interestingly, a higher portion of the COVID-19 OPC collective could be stabilised (39.3% vs. 16.9%), which differs significantly from the regular OPC collective.

However, based on these results we can not conclude whether OPC services are able to provide equivalent care to patients with or without COVID-19. Further studies regarding effectiveness of APC programs and OPC treatment in this special collective should be considered.

Our study further shows that the majority of nursing homes did not offer structural palliative care services during the COVID-19 pandemic in 2020 although they were one of the most frequent places of death. At the same time, only a minority (40.0%) of the surveyed nursing homes had structured ACP systems in place. This demonstrates that palliative care services were not generally available in nursing homes at the beginning of COVID-19 pandemic, even though the German public health insurance system financially supported their implementation [22]. Future studies must investigate whether the COVID-19 pandemic has changed these circumstances.

Therefore, our data underscores a strong need for the expansion of palliative care structures in German nursing homes. A well-structured and trained palliative care service for nursing home residents is needed to reduce unwanted and unnecessary hospital admissions. This could be achieved by creating more awareness for the patients ACP at the nursing home and by critically evaluating whether a multi-morbid resident would benefit from an emergency hospitalisation. This prehospital evaluation also could be helpful to avoid overburdening of healthcare systems during a pandemic. Technologies such as telemedicine also could be helpful, specifically in cases of infectious diseases and entry restrictions in nursing homes [23–25]. Interestingly, the majority of referrals of COVID-19 patients to our OPC team came from general practitioners (75.0%). These physicians usually are in very close contact with their patients and often able to assess their wishes, including those of patients without a written advance healthcare directive. Nevertheless, our data shows that those patients treated by our OPC service had an advance healthcare directive in 75.0% of all cases. This demonstrates that the outpatient COVID-19 collective was more likely to make conscious decisions about end-of-life medical care in comparison to the control collective (61.0%). This finding was not statistically significant, but it hints at the decisive role of general practitioners in advising patients and their relatives towards OPC. Even in a pandemic, the main motive for a hospitalisation or the decision towards OPC treatment should be according to the patient-will. Additionally, OPC services are capable of not only treating patients in their familiar surroundings but also advising and training their caregivers or families. This gives them a key role not only in conducting palliative care, but also in having a consulting role concerning the decision for or against hospitalisation.

An important difference when comparing the COVID-19-confirmed and non-COVID-19 OPC collectives was the number of patients with a cancer comorbidity. The number was significantly lower in the COVID-19-confirmed collective than in the non-confirmed collective (39.3% vs. 66.6%). This circumstance also possibly indicates the growing importance and knowledge about OPC not only for cancer patients but throughout the German health care system.

## Conclusions

Overall, our study highlights the potential of OPC services during a worldwide pandemic and it is the first to characterise this particular OPC collective of COVID-19 patients.

This study also demonstrates an urge for future development and enhancement of OPC structures in German nursing homes. We suggest implementing OPC services into national pandemic preparedness plans [26] and postulate that OPC has the potential to reduce unwanted hospitalisations, relieving conventional healthcare structures. Finally, our study points out the importance of expanding research on OPC patient collectives without cancer history in order to improve OPC services for non-oncologic multi-morbid patients.

## Supplementary Information

**Additional file 1.**

## Data Availability

All data generated or analysed during this study are included in this published article and its supplementary information files.

## Abbreviations

COVID-19: Corona virus disease 2019
OPC: Outpatient palliative care
ACP: Advance care planning
ECOG: Index of Eastern Cooperative Oncology Group
SGB V: Sozialgesetzbuch V (social law book V)
ICU: Intensive care unit
Avg: Average
SD: Standard deviation
vs.: Versus
